# A retrospective analysis of conditional power assumptions in clinical trials with continuous or binary endpoints

**DOI:** 10.1186/s13063-023-07202-6

**Published:** 2023-03-22

**Authors:** Julia M. Edwards, Stephen J. Walters, Steven A. Julious

**Affiliations:** 1 School of Health and Related Research, The University of Sheffield, 30 Regent Street, Sheffield, S1 4DA UK; 2 Intensive Care National Audit and Research Centre (ICNARC), 24 High Holborn, London, WC1V 6AZ UK

**Keywords:** Conditional power, Adaptive designs, Futility, Sample size re-estimation, Clinical trials

## Abstract

**Background:**

Adaptive clinical trials may use conditional power (CP) to make decisions at interim analyses, requiring assumptions about the treatment effect for remaining patients. It is critical that these assumptions are understood by those using CP in decision-making, as well as timings of these decisions.

**Methods:**

Data for 21 outcomes from 14 published clinical trials were made available for re-analysis. CP curves for accruing outcome information were calculated using and compared with a pre-specified objective criteria for original and transformed versions of the trial data using four future treatment effect assumptions: (i) observed current trend, (ii) hypothesised effect, (iii) 80% optimistic confidence limit, (iv) 90% optimistic confidence limit.

**Results:**

The hypothesised effect assumption met objective criteria when the true effect was close to that planned, but not when smaller than planned. The opposite was seen using the current trend assumption. Optimistic confidence limit assumptions appeared to offer a compromise between the two, performing well against objective criteria when the end observed effect was as planned or smaller.

**Conclusion:**

The current trend assumption could be the preferable assumption when there is a wish to stop early for futility. Interim analyses could be undertaken as early as 30% of patients have data available. Optimistic confidence limit assumptions should be considered when using CP to make trial decisions, although later interim timings should be considered where logistically feasible.

**Supplementary Information:**

The online version contains supplementary material available at 10.1186/s13063-023-07202-6.

## Introduction

Clinical trials require a minimum number of subjects in order to answer the research question at hand. In addition to likely estimates of the effect size, key components of the sample size calculation are statistical significance/type I error (probability of incorrectly rejecting the null hypothesis) and statistical power (the probability of correctly detecting a significant result of a given magnitude) [1]. Many trials recruit at least the minimum required number of subjects and analyse the results after recruitment is complete.

However, trialists may wish to incorporate an interim analysis for the purpose of making an adaptation to a trial, such as stopping a trial for either efficacy or futility or modifying the sample size in a bid to maintain statistical power. Such decisions may be based on conditional power (CP), the probability of rejecting the null hypothesis at the final analysis, given the data observed at the interim analysis and a future treatment effect for remaining patients [2, 3]. Alternative designs, such as group sequential designs have well established methods including “spending” some type I error at an interim analysis in order to formally perform hypothesis tests on the accrued data and potentially stop for efficacy and/or futility.

Regardless of the assumptions used in the CP equation, it is impacted by the accruing data. An interim analysis very early on in the trial can have huge impacts on trial decisions but may be based on a very different treatment effect size than that which may have been observed at the end of the originally planned trial. Additionally, patients recruited at the start of the trial may be inherently different to those recruited at the end. New emerging medical guidance, opening additional sites in a bid to finish recruitment on time, or even protocol amendments such as a change in eligibility criteria are some of the reasons these patient populations may differ.

There has been some debate in statistical literature over the choice of future treatment effect assumption [4–6], and each can yield very different results. Therefore, it is critical that these assumptions are understood by trialists wishing to base key decision making on designs using conditional power. This publication presents results from a re-analysis of 14 real-world published trials, with the purpose of investigating the impact of four future treatment effect assumptions in the conditional power equation.

### Aims and objectives

The main objective of this paper was to use real-world trial data to investigate four possible future treatment effect assumptions used in the conditional power equation and to make recommendations for researchers implementing such designs in practice. Additionally, the timing of the interim analysis is explored through evaluating stability of the estimate during the trial.

## Methods

Data was obtained from 14 real-world clinical trials from publicly funded studies in the UK and industry companies participating in the Clinical Study Data Request (CSDR) platform (www.clinicalstudydatarequest.com). Re-analysis of real-world data allows observation of estimate instability during patient accrual, recruitment pace of both sites and patients, and time to availability of primary outcome data.

Trials were chosen to provide a range of sample sizes (from *n* = 86 to *n* = 2249) and length of time to follow-up for the primary endpoint (from 7 days to 2 years). Trials were restricted to primary endpoints with either continuous or binary data, as defined in the original analysis by the trial team. For standardised comparisons of trials with binary endpoints, outcome measures were converted to an odds ratio (OR). Additionally, models adjusted for variables used in the final analysis of the original trial data.

Applications for data use was made over a 12-month period, and full details can be found in the online Supplementary material. Initially, 13 publicly funded trials were identified from two National Institute for Health Research (NIHR) journals, where it is strongly encouraged to make trial data available. An additional five were requested internally through the University of Sheffield. An additional 12 industry funded trials meeting these criteria were requested from the CSDR platform. Between initial application and final data sharing agreements in place, three companies left the CSDR platform and a further three datasets were not received by the cut-off date. Data from co-primary or secondary outcomes were used where available to maximise data usage, resulting in a total of 21 distinct outcomes from 14 trials available for this re-analysis. All datasets made available for analysis were from non-adaptive fixed sample size designs.

The stability of the estimate (mean difference for continuous endpoints; log(OR) for binary endpoints) was investigated in order to understand when might be the earliest time that an interim analysis could take place, in terms of patients with data available. The end result of the original trial analysis was taken as the “true” treatment effect. Treatment estimates after every 10–20 patients (depending on the size of the trial) with data available were calculated using the same order of recruitment as in the original trial (‘original sequential order’). This was repeated using the reverse sequential order of patients as a comparison of any potential bias between patients recruited at the very start of the trial, compared to those recruited at the end. Trial participants were then randomly re-ordered 1000 times, and again treatment estimates were calculated. The median treatment estimate of the 1000 random reordering of patients was plotted alongside the original and reverse sequential orders, with 97.25% and 2.5% and 75% and 25% quantiles.

Conditional power is the probability of rejecting the null hypothesis at the final analysis, given the current data, and is calculated using the following formula [7]:

$$CP\left(n|{z}_1\right)=1-\varPhi \left\{\frac{z_{\alpha}\sqrt{n}-{z}_1\sqrt{n_1}}{\sqrt{\left(n-{n}_1\right)}}-\frac{\overset{\sim }{d}\sqrt{n-{n}_1}}{\sqrt{2{\hat{\sigma}}_{obs}^2}}\right\},$$

where *z*_1_ is the observed test statistic at the interim analysis, ${\hat{\sigma}}_{obs}^2$is the observed variance of the outcome at the interim analysis which is assumed the same in both groups, $\overset{\sim }{d}$ is the assumed future treatment difference, *z*_*α*_ is the critical value for the final analysis, *Φ*() is the standard normal cumulative distribution function, *n*_1_ is the total sample size at the interim analysis, and *n* denotes the total sample size at the planning stage of the trial.

The most commonly used CP assumptions are (1) current trend ($\overset{\sim }{d}={\hat{d}}_{obs}$; assuming the data observed so far is likely to continue for the duration of the trial), sometimes referred to as ‘observed conditional power’ [8] and (2) the hypothesised treatment effect ($\overset{\sim }{d}={d}_{plan}$; assuming the hypothesised treatment effect used in the original sample size calculation), sometimes referred to as ‘assumed conditional power’ [8]. There is criticism in the literature regarding the current trend assumption, due to high variability early in the trial duration and potentially yielding an unstable estimate of conditional power values [6]. An alternative recommendation from the literature [7, 9] is based on optimistic confidence limits of the observed treatment effect, being the single optimistic value of the two confidence limits defined by $\overset{\sim }{d}={\hat{d}}_{obs}\pm {Z}_{1-\frac{p}{2}}\sqrt{\frac{2{\hat{\sigma}}_{obs}^2}{n_1}}$, where ${Z}_{1-\frac{p}{2}}$represents the $\left(1-\frac{p}{2}\right)$ percentage point of a standard normal distribution.

Two further treatment effect assumptions were investigated in the re-analysis based on (1) the 80% optimistic confidence limit (*p* = 0.2) and (2) the 90% optimistic confidence limit (*p* = 0.1)

CP was calculated using each of the four chosen future treatment effect assumptions after every patient with completed follow-up data and plotted from patient 20 onwards. A futility boundary of 10% conditional power value is considered for CP assumption comparison in this re-analysis, although other values may be chosen, with boundaries between 10 and 40% being observed in the literature [2, 9]. The smaller value has been chosen for this re-analysis to accommodate sample size re-estimation rules based on CP decisions, with alterations to sample size often occurring below 40% CP [3, 6]. CP is calculated after every patient for illustrative purposes of CP during the trial progression. However, it should be noted that in reality this is calculated just once.

Whilst observing CP of each trial as close to the original setting as possible is interesting, it is difficult to make direct comparisons between trials, as the magnitude of difference between the planned effect and the observed end result varied from trial to trial. For more appropriate comparisons, the data from each trial were transformed such that the observed treatment effect at *n* patients: (1) matched that planned in the original protocol and sample size calculation, (2) slightly smaller than planned (2/3 of the original planned effect), (3) much smaller than planned (1/3 of the original planned effect), and (4) zero.

For continuous endpoints, each primary outcome value was multiplied by some constant such that the observed standard deviation in the trial equalled that used in the original sample size calculation. Following this, another constant value was added to each intervention arm patient, such that the mean difference matched that in the original sample size calculation. This procedure was repeated, altering the constant added to the intervention arm in order to reach the desired end treatment effect (as planned, smaller than planned, or zero)

For binary endpoints, the model coefficient, or log(OR), was multiplied by some constant such that the standard error after n recruited patients matched that used in the original sample size calculation. Another constant was added to the transformed coefficients in the intervention group to ensure the log(OR) matched that used in the original sample size calculation. Again, the procedure was repeated, altering the constant added to the intervention arm in order to reach the desired end treatment effect (as planned, smaller than planned, or zero)

Conditional power lines are assessed using the following objective criteria:

- Given a statistically significant finding at the original *n* patients, conditional power values should be high and not fall into the futility boundary.
- If the findings at *n* patients are lower than planned, a low conditional power throughout would be ideal, potentially triggering early termination of the trial for futility.

Ethics approval from the University of Sheffield for the secondary use of patient data was obtained on the 16 August 2019 (reference number 030485).

## Results

### Case studies

**Fig. 1:**
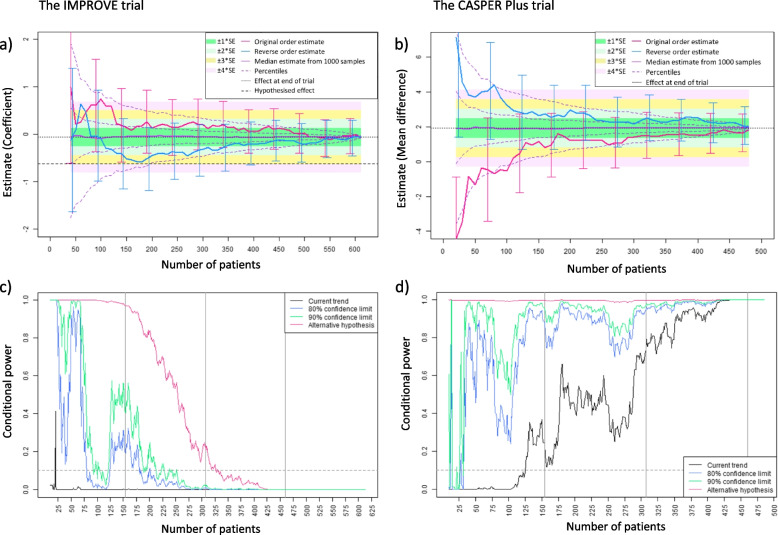
**a**, **b** Stability of the treatment estimate during trial progression for IMPROVE (log(OR)) and CASPER Plus (mean difference) studies respectively, showing estimates and 95% confidence intervals given patient enrolment occurred in original sequential order, reverse order, and simulated random re-ordering for 1000 random samples (median and 2.5, 25, 75, 97.5 percentiles). **c**, **d** Conditional power curves for the original trial data for IMPROVE and CASPER Plus respectively, calculated using four assumptions of future treatment effect. A 10% futility boundary is also shown (dashed line)

**Fig. 2:**
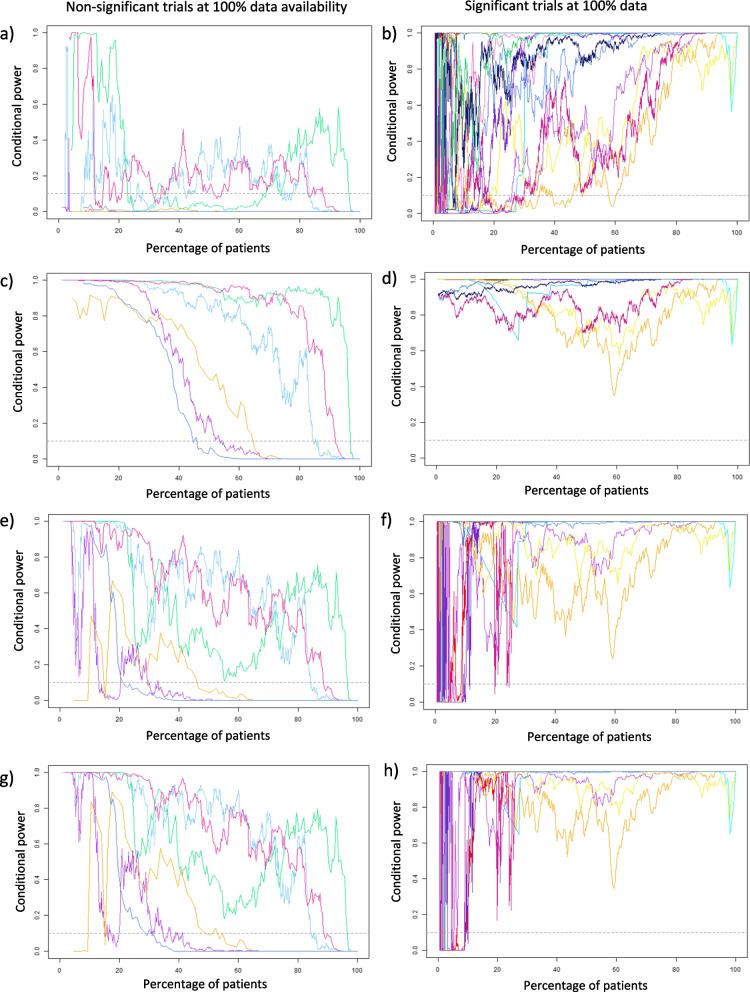
Conditional power curves for all re-analysed trials using the current trend assumption (**a**, **b**), the hypothesised assumption (**c**, **d**), the 80% optimistic confidence limit assumption (**e**, **f**), and the 90% confidence limit assumption (**g**, **h**), split by statistical significance in the original analysis: non-significant (**a**, **c**, **e**, **g**) and significant (**b**, **d**, **f**, **h**)

**Fig. 3:**
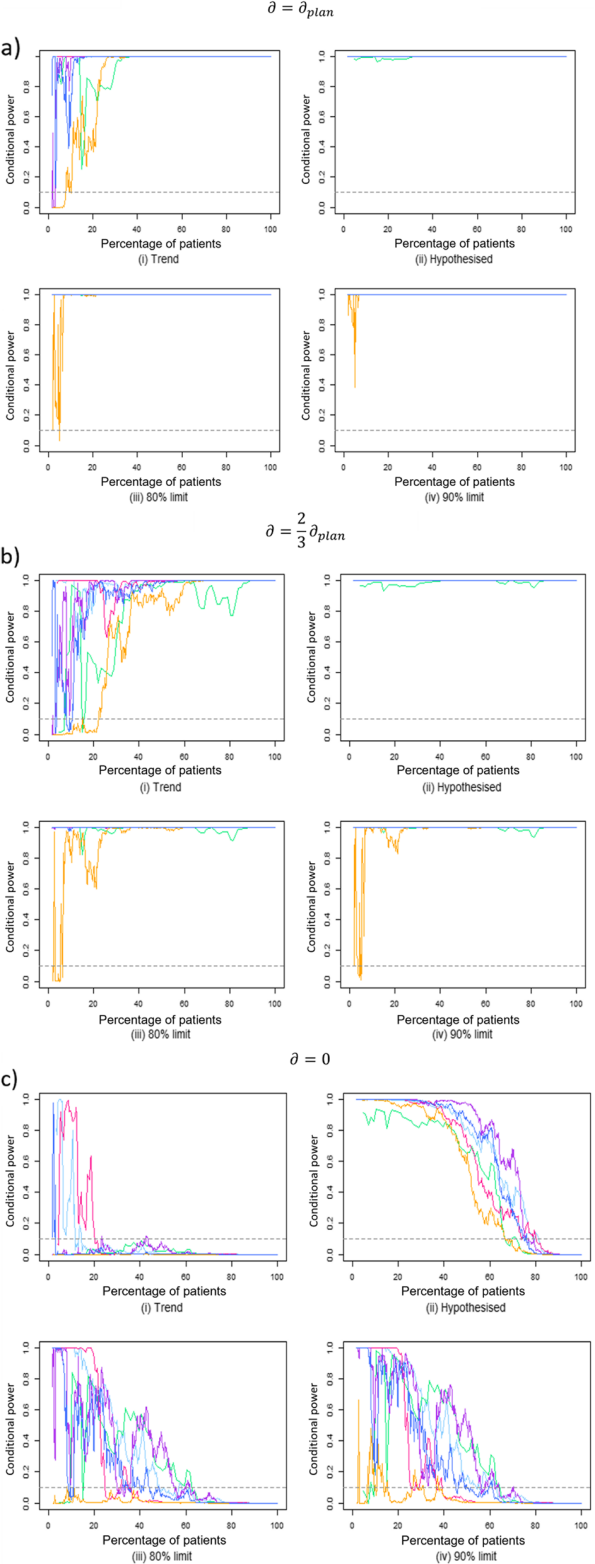
Conditional power curves for trials with continuous outcome data following data transformations such that observed final estimate equals: **a ***∂* = *∂*_*plan*_; **b **$\partial =\frac{2}{3}{\partial}_{plan}$; **c ***∂* = 0. For each data transformation scenario, four future treatment effect assumptions are used in the conditional power calculation

**Fig. 4:**
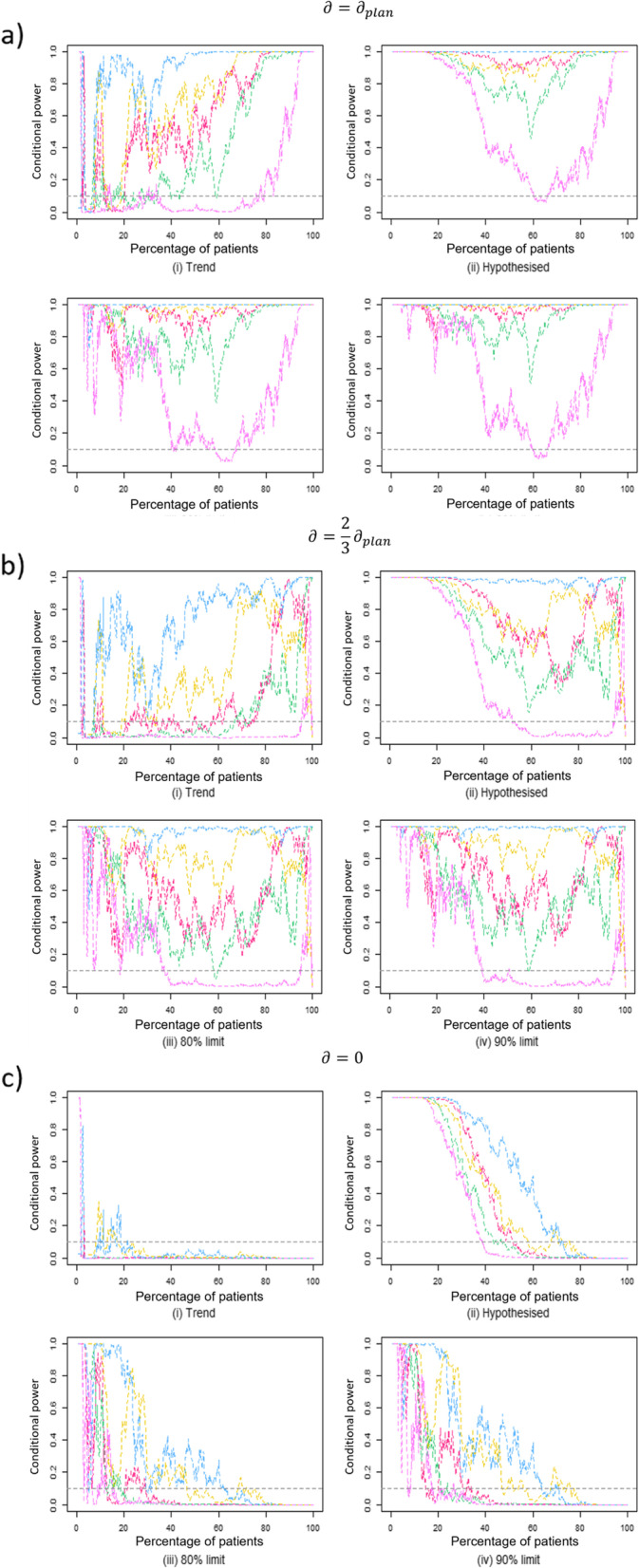
Conditional power curves for trials with binary outcome data following data transformations such that observed final estimate equals: **a ***∂* = *∂*_*plan*_; **b **$\partial =\frac{2}{3}{\partial}_{plan}$; **c ***∂* = 0. For each data transformation scenario, four future treatment effect assumptions are used in the conditional power calculation

In-depth results from two case studies (one continuous and one binary endpoint; one with a statistically significant result in the original trial and one showing no significant difference) are presented in Fig. 1. Additionally, all trial results are presented in Figs. 2, 3, and 4 and Table 1.

**Table 1:** Details of trials included in the retrospective analysis including reference, sample size, primary outcome measure and collection time, number of sites, and time in days of patient recruitment period; by relative length of time to primary outcome data collection

**Continuous outcomes; publicly funded**			
**“Small”**	**Trial 1** [13] FAST INdiCATE *n* = 288 Upper limb functionality (ARAT score) at 6 weeks 3 sites; 1199 days	**Trial 2** [14] SELF *n* = 86 Pain score (SPADI) at 3 months 3 sites; 477 days	**Trial 3** [15] Acupuncture *n* = 241 Pain score (SF-36) at 12 months 16 sites; 549 days
**“Large”**	**Trial 4B** [16] CASPER *n* = 705 Depression severity (PHQ-9) at 4 weeks^b^ 4 sites; 761 days	**Trial 5** [11] CASPER Plus *n* = 485 Depression severity (PHQ-9) at 4 months 4 sites; 705 days	**Trial 4A** [14] CASPER *n* = 705 Depression severity (PHQ-9) at 12 months 4 sites; 761 days
**Continuous outcomes; industry**			
**“Small”**	-	**Trial 6** [17] Epilepsy *n* = 133 Cognitive function at 19 weeks 3 sites; 945 days	-
**“Large”**	**Trial 7A** [18] Flu vaccine (A/H1N1) *n* = 2249 Geometric mean titer ratio^a^ at 28 days 38 sites; 12 days	**Trial 7B** [18] Flu vaccine (A/H3N2) *n* = 2249 Geometric mean titer ratio^a^ at 3 months^b^ 38 sites; 12 days	**Trial 7C** [18] Flu vaccine (B1) *n* = 2249 Geometric mean titer ratio^a^ at 1 year^b^ 38 sites; 12 days
**Binary outcomes; publicly funded**			
**“Small”**	**Trial 8** [10] IMPROVE *n* = 613 Mortality at 30 days 31 sites; 1380 days	**Trial 9** [19] Corn plasters *n* = 202 Resolution at 3 months 7 sites; 779 days	**Trial 10** [20] AMAZE *n* = 352 Return to sinus rhythm at 2 years 311 sites; 2016 days
**“Large”**	-	**Trial 12** [21] RATPAC *n* = 2243 Successful hospital discharge at 3 months 6 sites; 490 days	**Trial 11** [22] 3MG *n* = 1109 Admission to hospital at 12 months 25 sites; 1427 days
**Binary outcomes; industry**			
**“Small”**	**Trial 13** [23] Nasal sprays *n* = 300 Patient preference; < 1 h 12 sites; 765 days	**Trial 14A** [24] Mencevax (A) vaccine *n* = 296 SBA titer ≥ 128^a^ at 1 month 2 sites; 199 days	**Trial 14B** [24] Mencevax (C) vaccine *n* = 296 SBA titer ≥ 128^a^ at 1 year^b^ 2 sites; 199 days
**“Large”**	**Trial 7D** [18] Flu vaccine (A/H1N1) *n* = 2249 Seroconversion rate^a^ at 28 days 38 sites; 12 days	**Trial 7E** [18] Flu vaccine (A/H3N2) *n* = 2249 Seroconversion rate^a^ at 3 months^b^ 38 sites; 12 days	**Trial 7F** [18] Flu vaccine (B1) *n* = 2249 Seroconversion rate^a^ at 1 year^b^ 38 sites; 12 days

^a^Co-primary outcome

^b^Re-imagined time point of primary outcome data collection

The IMPROVE trial was designed to detect a risk difference of 14.3% in mortality at 30 days (binary endpoint) with 94% power. The trial recruited 613 patients from 31 sites to two surgical interventions for abdominal aortic aneurysm between September 2009 and July 2013. However, the IMPROVE trial did not find a statistically significant result between the two groups after adjusting for age, sex, and Hardman index (OR = 0.92, 95% CI (0.66, 1.28), *p* = 0.62) [10].

The CASPER Plus trial aimed to detect a clinically important effect size of 0.35 using a depression severity questionnaire score (PHQ-9) at 4 months from randomisation (continuous endpoint). Between September 2012 and August 2014, 485 patients were randomised from 4 sites, and the trial found a significant reduction in depression score after adjusting for baseline questionnaire scores (MD = 1.92, 95% CI = (0.85, 2.99), *p* < 0.001) [11].

Figure 1 shows the results of the re-analysis of the original trial data for the IMPROVE trial and the CASPER Plus trial. Figure 1a and b show the stability of the estimate using the original sequential order of patients (pink), the reverse sequential order (blue), and the median of 1000 random orders (purple), calculated after every 10 patients, starting at patient 40 due to high variability. Four boundaries are chosen to represent potential estimate stability in terms of multiples of SE away from the original trial estimate (here, assumed the ‘true’ treatment effect): ± 1*SE (dark green), ± 2*SE (pale green), ± 3*SE (yellow), and 4*SE (pale pink). The CASPER Plus trial provided hypothesised values as a treatment effect as opposed to hypothesised mean difference and standard deviation and is therefore omitted from Fig. 1b.

The IMPROVE trial original order estimate lies outside even the ± 4*SE boundary at 100 patients with available data (18% through the trial) and first reaches the ± 1*SE boundary at 160 patients (26% through the trial). The estimate fluctuates and does not remain in the smallest boundary considered until 460 patients (75% through the trial). The reverse order estimate always lies within the ± 4*SE boundary, first reaches the ± 1*SE boundary at 40 patients (just 7% through the trial), and remains within the smaller boundary from patient 350 onwards (57% through the trial).

The CASPER Plus trial original order estimate starts much lower than that observed at the end of the original analysis of the trial. However, a steady increase means the estimate reaches the ± 4*SE, ± 3*SE, ± 2*SE, and ± 1*SE boundaries by 110, 120, 130, and 180 patients respectively. From 300 patients onwards (62% through the trial), the estimate remains in the smaller ± 1*SE boundary. The reverse order estimate, however, takes longer to reach the ± 1*SE boundary (240 patients, 49% through the trial) and to remain within this boundary (410 patients onwards, 85% through the trial).

Figure 1c and d show the conditional power lines for the two trials calculated after every consecutive patient with data available. Four lines represent the four different future treatment effect assumptions; current trend (black), hypothesised (red), 80% confidence limit (blue), and 90% confidence limit (green).

Using the IMPROVE trial data, conditional power under the observed current trend assumption starts very low, and other than one spike around 20 observed patients, remains below 0.01 throughout. On the other hand, conditional power under the hypothesised treatment effect starts at 100% for the first 100 patients and gradually decreases from this point forward until reaching zero by patient 430. The optimistic confidence limits of the observed current trend result in large fluctuations early in the trial, with a large second spike around 110–200 patients. Both optimistic limit lines settle to near zero by the 50% data availability time point.

The conditional power line under the observed current trend assumption using the data from the CASPER Plus trial starts at zero and gradually increases to 100% around 430 patients (89% data availability). CP under the hypothesised assumption is consistently high throughout the trial duration. Similarly to the IMPROVE trial, optimistic confidence limits CP values lie between the CP values observed under the current trend and hypothesised assumptions.

### Overview of all trials (original data)

Collating the retrospective analysis results from all 21 cases studies, CP lines are presented for each future treatment effect assumption. To evaluate CP lines, the trials have been split into significant vs non-significant findings of the original trial, and evaluated using the objective criteria (Fig. 2).

The CP values generally have the lowest conditional power under the observed current trend assumption for trials with either significant or non-significant final findings at *n* patients. Whilst the trials with non-significant findings may have benefitted from stopping early for futility, almost all trials with significant final findings would have stopped for futility before a 30% interim analysis. Even at the 60% interim time point, one trial (orange) would have stopped for futility, despite a significant finding at the original *n* patients. Trialists using the observed current trend effect should do so with extreme caution if wishing to present early findings, as results could change substantially between early interim time points and final analyses.

CP values under the hypothesised effect assumption start high and is slow to decrease for trials with non-significant findings. Only one trial reaches a 10% futility boundary by the halfway mark, and three of the six trials remain above this boundary until at least 85% through the original trial duration. Incorporation of a futility boundary would therefore be unlikely to ‘save’ any additional patients as they would likely have all been recruited by this time point with data available. For trials with significant findings, CP values are highest under the hypothesised assumption.

The CP lines using the 80% and 90% optimistic confidence limit assumptions see very large fluctuations of CP at the very start of the trial, particularly in those with significant findings in the original analysis. This rapidly changing CP fluctuations largely stops by 15% original trial duration. After the 25% interim time point with data available, no trial with a significant finding in the original analysis would have stopped for futility in the re-analysis. By 30% original trial duration, most trials see high conditional power values, with the exception of one trial, albeit not as severe a dip as under the current trend.

### Data transformations

Three scenarios are presented following transformation of the data such that the end observed effect is as planned, smaller than planned, and zero. Other values have been investigated and are included in the online Supplementary material. The same four assumptions of the future treatment effect investigated for the retrospective case studies are compared for each scenario of end treatment effect observed at *n* patients.

One trial with a binary endpoint observed results much higher than that planned (pink). Therefore, the trial results saw dramatic decreases in the data transformation, but is included in the data analysis for completeness.

$$\partial ={\partial}_{plan}$$

Similarly to the re-analysis of original trials, when the treatment effect observed at the end of the trial is exactly as planned, conditional power lines should be high. The end result for all trials would be statistically significant due to observing the planned treatment effect at 100% patient recruitment.

For continuous trials (Fig. 3a) using the observed current trend assumption, the conditional power lines fluctuate early on but reach approximately 100% before 40% through the originally planned trial. For binary trials (Fig. 4a) under the current trend assumption, however, conditional power lines are slow to reach the high values. A slow increase in conditional power could be problematic for designs trying to stop for efficacy at this point given that *∂* = *∂*_*plan*_ or for basing a decision to modify sample size.

Under the hypothesised assumption, continuous trials are consistently high throughout trial duration, which may be beneficial for stopping for efficacy. Conditional power is high for most trials with binary endpoints, with the exception of one trial that falls into the 10% futility boundary between 60 and 70% data available.

Both optimistic limits see early fluctuations in one trial with a continuous endpoint before 15% through the trial. From this point, however, all lines are approximately 100% for the remainder of the trial. For trials with binary endpoints, optimistic confidence limits follow a similar pattern to the hypothesised assumption, with the same 60-70% trial duration dip into the 10% futility boundary zone.

$$\partial =\frac{2}{3}{\partial}_{plan}$$

This scenario illustrates when the treatment effect observed at the end of the trial is smaller than planned. It should be noted that an effect size of two thirds planned could be statistically significant, but may not be clinically relevant in every trial, particularly if *∂*_*plan*_ for the original sample size calculation was chosen as the minimum clinically important difference.

The conditional power values using any of the four investigated assumptions for outcomes with continuous data is still predominantly > 80% (Fig. 3b). Prior to 20% trial duration, however, the current trend assumption falls below 10% conditional power values for four trials, compared to one trial prior to 10% trial duration using optimistic confidence limits, or none using the hypothesised assumption.

Trials with binary outcome data (Fig. 4b) show much more variation in conditional power, with values fluctuating greatly throughout trial duration using any of the four investigated future treatment effect assumptions. Using the current trend assumption yields, the most trial duration spent below 10% conditional power (median duration 51.5%, IQR (14–65%)). The high variation could be due to $\frac{2}{3}{\partial}_{plan}$ being too small an effect, especially if the original sample size calculation is based on the minimum clinically important difference

$$\partial =0$$

When the treatment effect observed at the end of the trial is zero (i.e. no difference between groups), the conditional power lines are below the 10% futility bound under the current trend assumption for both binary and continuous endpoints by a 20% interim time point. No trial result is statistically significant at 100% recruitment.

Under the hypothesised treatment effect assumption, however, the first trial to fall below this boundary is 38% trial duration for binary endpoints (Fig. 4c) and not until 67% for continuous endpoints (Fig. 3c). All trials drop below this boundary by 85% trial duration under this assumption, despite no difference in treatments.

Again, the conditional power values using an optimistic confidence limit assumption fall somewhere between the current trend and hypothesised assumptions. Whilst not falling below the 10% futility boundary as early as seen under the current trend assumption, almost all trials are within this boundary by the 60% mark.

## Discussion

After considering conditional power values under these scenarios, the current trend appears to be the most favourable assumption to use when the true treatment effect is very small or no difference at all if wishing to stop for futility due to earlier timings of low observed CP values. However, the current trend assumption does not perform as well in terms of CP when the treatment effect is as planned or smaller than that planned, observing huge fluctuations, and the lowest CP values of the four assumptions across multiple trials (Figs. 3b and 4b). For this reason, trials wishing to stop early for futility may wish to use the current trend assumption and could see this as early as 20–30% trial duration under the current trend assumption (Figs. 3c and 4c) using a 10% futility boundary. However, trialists should note the early fluctuations observed with < 20% trial duration even when *∂* = *∂*_*plan*_ and may wish to consider a later interim timing, when we have more information.

This effect is switched, however, when using the hypothesised assumption for the future treatment effect; performing well when the treatment effect is as planned (observed high CP values throughout) but slower than the current trend to reach a 10% futility boundary when the true treatment effect is zero. For this reason, designs making decisions based on conditional power that do not wish to stop early for futility may wish to use the hypothesised assumption for the future treatment effect for the conditional power calculations. However, should there be no effect to observe in reality, this design would need a late interim analysis (approx. 80% trial duration/information) in order to stop early for futility.

On the other hand, the optimistic confidence limit assumptions could offer a compromise between the two current accepted assumptions used in practice and could benefit designs making additional decisions at an interim analysis, such as those wishing to modify sample size to maintain power [3].

Given that it is unknown at the planning stage whether the trial will have an effect close to that planned, or no difference at all, the optimistic confidence limit assumptions could again provide an alternative to the current trend and hypothesised assumptions. Whilst it is hoped at the start of a superiority trial that a difference between groups may be observed, it is pertinent to consider the option of seeing no effect and adding a safety net to stop prior to the original planned sample size. A review of 107 publicly funded UK trials publishing results between 2006 and 2016 found that the median standardised target effect size was 0.3 (IQR: 0.2–0.38), whereas the median standardised observed effect size was 0.11 (IQR: 0.05–0.29) [12]. Therefore, an optimistic confidence limit with an interim analysis timing between 60 and 70% could be considered.

At all instances of investigated delta, trials with binary endpoints see highly variable CP values. Due to the conversion of outcome measures to an OR for standardised comparisons, variance may have been affected and contributing to the high level of fluctuations observed in CP values.

One limitation of this paper is that there is no simulation work included. The true value of the end treatment effect has been assumed to be that observed in the original trial data, whereas simulation results would allow the estimation of the true treatment effect. However, a particular strength of this paper is the use of real-world trial data. Time of patient and site recruitment, inherent differences in patient populations between the start and end of a trial, and availability of primary outcome data are all challenging to simulate. Retrospective analysis of trial data allows the observation of conditional power values with realistic inputs of trial progression.

This paper illustrates the importance of thinking about the future treatment effect assumption used in the conditional power calculation prior to start of trial, as assumptions can yield very different results. Additionally, trialists or data monitoring committees may wish to consider more than one conditional power value in which to base a decision. However, designs with formal decisions based on conditional power, such as a sample size re-estimation, may require one pre-specified assumption due to regulatory concerns [3].

## Conclusion

When there is a wish to stop early for futility, the current trend assumption with an interim analysis after around 30% of participants have been recruited and follow-up up to the primary outcome, time point might be considered.

Optimistic confidence limits may provide a good compromise between the hypothesised and current trend current assumptions used in the conditional power equation, especially when there is uncertainty over what the trial may demonstrate. However, a later interim analysis timing of around 60–70% data availability should be considered using this assumption where logistically feasible.

## Supplementary Information

**Additional file 1: Supplementary Figure 1.** Flowchart of the data request process for industry data. **Supplementary Figure 2.** Flowchart of the data request process for publicly funded trials. **Supplementary Figure 3.** Conditional power curves for trials with continuous outcome data following data transformations such that $\partial =\frac{1}{3}{\partial}_{plan}$. For each data transformation scenario, four future treatment effect assumptions are used in the conditional power calculation. **Supplementary Figure 4.** Conditional power curves for trials with binary outcome data following data transformations such that $\partial =\frac{1}{3}{\partial}_{plan}$. For each data transformation scenario, four future treatment effect assumptions are used in the conditional power calculation.

## Data Availability

All trial datasets are available upon request to the original trial team for publicly funded trials and through the Clinical Study Data Request (CSDR) platform (www.clinicalstudydatarequest.com) for industry trials. A full list of trials is included in Table 1.
