# Supplementary material for: A retrospective analysis of conditional power assumptions in clinical trials with continuous or binary endpoints
Source: Trials. 2023 Mar 22;24:215. doi: 10.1186/s13063-023-07202-6 (PMC10035140; doi:10.1186/s13063-023-07202-6)
Supplement: Supplementary file 1 — Additional file 1: Supplementary Figure 1. Flowchart of the data request process for industry data. Supplementary Figure 2. Flowchart of the data request process for publicly funded trials. Supplementary Figure 3. Conditional power curves for trials with continuous outcome data following data transformations such that $\partial =\frac{1}{3}{\partial}_{plan}$∂=13∂plan. For each data transformation scenario, four future treatment effect assumptions are used in the conditional power calculation. Supplementary Figure 4. Conditional power curves for trials with binary outcome data following data transformations such that $\partial =\frac{1}{3}{\partial}_{plan}$∂=13∂plan. For each data transformation scenario, four future treatment effect assumptions are used in the conditional power calculation. [file 13063_2023_7202_MOESM1_ESM.docx]

**Supplementary Figure 1:** Flowchart of the data request process for industry data

**
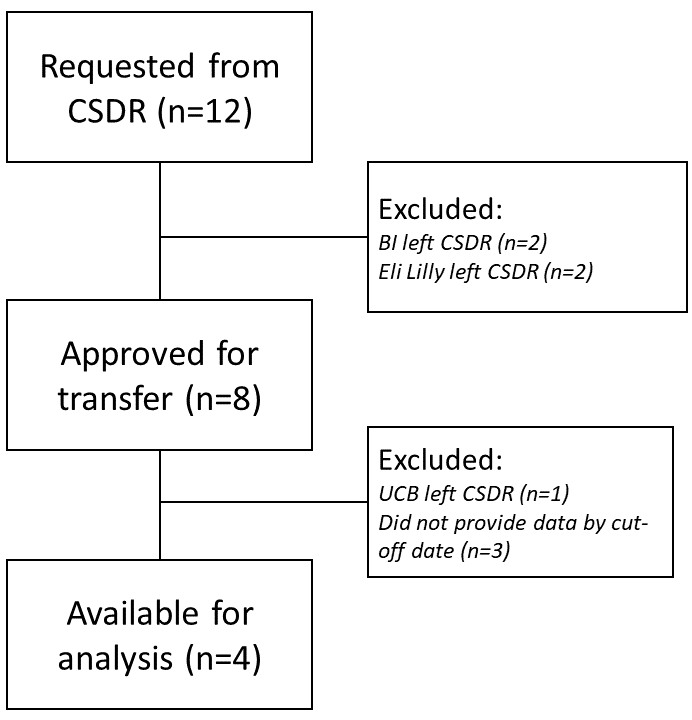
**

**Supplementary Figure 2:** Flowchart of the data request process for publicly funded trials

**
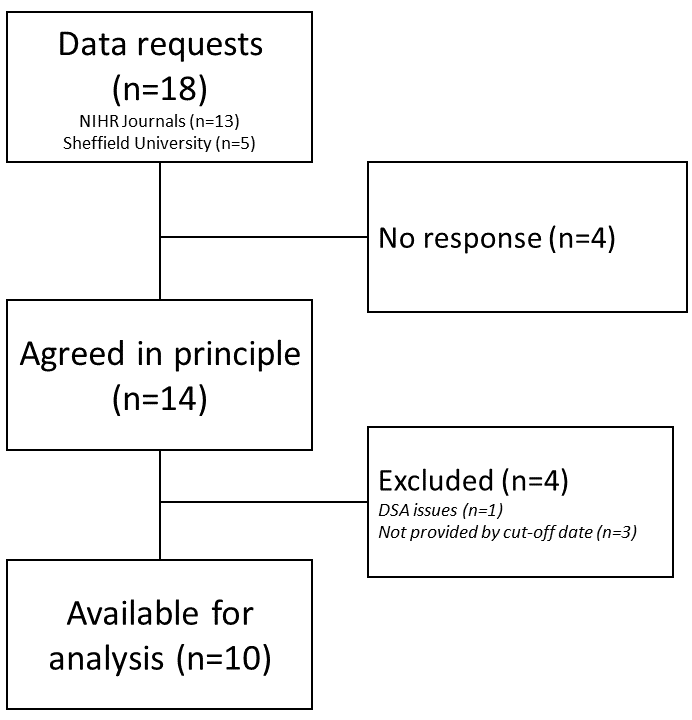
**

**Supplementary Figure 3:** Conditional power curves for trials with continuous outcome data following data transformations such that $\partial={\frac{1}{3}\partial}_{plan}$. For each data transformation scenario, four future treatment effect assumptions are used in the conditional power calculation

**
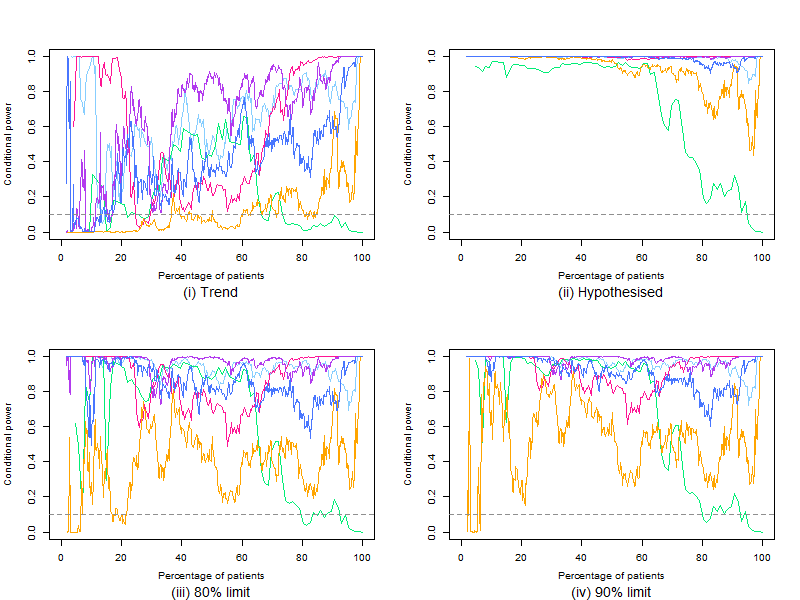
**

$$\partial=\frac{1}{3}\partial_{plan}$$

**Supplementary Figure 4:** Conditional power curves for trials with binary outcome data following data transformations such that $\partial={\frac{1}{3}\partial}_{plan}$. For each data transformation scenario, four future treatment effect assumptions are used in the conditional power calculation

**
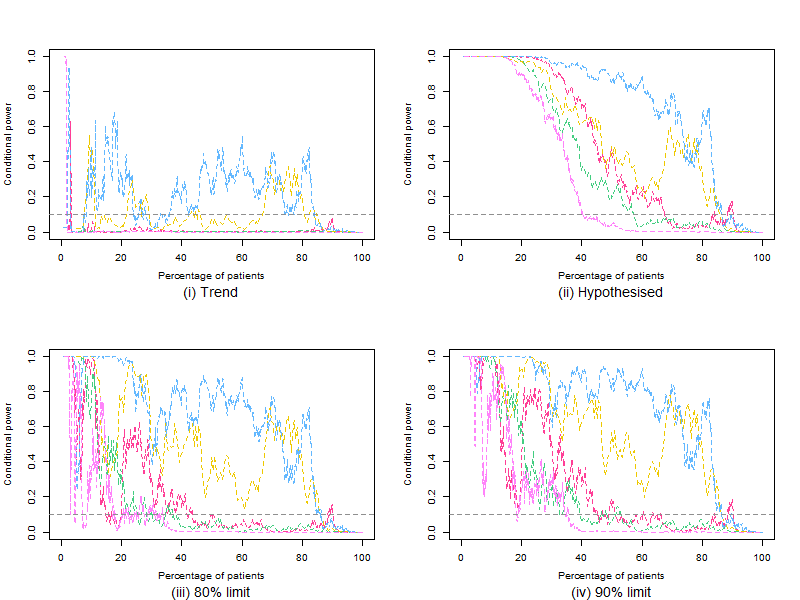
**

$$\partial=\frac{1}{3}\partial_{plan}$$
